# Pediatric Visceral Leishmaniasis Caused by *Leishmania infantum* in Northern Cyprus

**DOI:** 10.4269/ajtmh.16-0831a

**Published:** 2017-03-08

**Authors:** Pagliano Pasquale, Ascione Tiziana

**Affiliations:** 1 Department of Infectious Diseases, D. Cotugno Hospital, AORN Dei Colli, Naples, Italy. E-mail: ppagliano@libero.it; 2 Department of Infectious Diseases, D. Cotugno Hospital, AORN Dei Colli, Naples, Italy. E-mail: tizianascione@hotmail.com

Dear Sir:

Sayili and others^1^ report three cases of visceral leishmaniasis (VL) caused by *Leishmania infantum* (also called Mediterranean VL) occurring in northern Cyprus. They state that fever, splenomegaly, and hepatomegaly are the typical findings on physical examination and that marked anemia with pancytopenia, increased erythrocyte sedimentation rate, and high levels of C-reactive protein are the most common laboratory findings. Although these symptoms are very frequently associated with VL, they are nonspecific. We have the following concerns.

First, the authors do not report on the nutritional status of the children affected by VL or the time between symptom onset and diagnosis. These findings should be considered in children with fever of unknown origin and hepatosplenomegaly, because the suspicion of VL is greater in those with long-lasting fever and poor nutritional status.^2^

Second, in the Mediterranean area, chronic hepatitis is common, with prevalence as high as 1% of the population.^3^ The main signs of cirrhosis such as an enlarged liver and spleen, pancytopenia, and increased γ-globulin concentration can mask those of VL, sometimes suggesting liver disease decompensation rather than VL. In patients with cirrhosis, a positive VL serology can be sufficient to support a presumptive diagnosis and treatment. Of note, VL can worsen liver function of patients with cirrhosis, and liver function improves after treatment.^4^

Third, the authors do not clearly state that outside hyperendemic areas most cases of VL are diagnosed in those with impairment of cell-mediated immunity or receiving immunosuppressive therapy. In such patients, symptoms may be nonspecific, with overlap between those of the underlying disease and VL, with fever absent and pancytopenia and weight loss the only signs reported.^5^,^6^

In our opinion, the authors do not adequately emphasize that Mediterranean VL should be suspected in cases of fever of unknown origin in patients with chronic diseases, poor nutritional status, or receiving immunosuppressive drugs. Moreover, VL should be considered among the causes of unexplained liver decompensation in patients living with cirrhosis.
