# Erratum

**DOI:** 10.4269/ajtmh.965err

**Published:** 2017-05-03

In *Am J Trop Med Hyg 96:* 758 (https://doi.org/10.4269/ajtmh.16-0831a), there was an error in the author list. Pagliano Pasquale and Ascione Tiziana should have appeared as Pasquale Pagliano and Ascione Tiziana. The journal regrets the error.

